# PosturAll: A Posture Assessment Software for Children

**DOI:** 10.3390/bioengineering10101171

**Published:** 2023-10-08

**Authors:** Ana Beatriz Neves, Rodrigo Martins, Nuno Matela, Tiago Atalaia

**Affiliations:** 1 Instituto de Biofísica e Engenharia Biomédica, Faculdade de Ciências, Universidade de Lisboa, 1749-016 Lisboa, Portugal; nmatela@fc.ul.pt; 2 Escola Superior De Saúde Da Cruz Vermelha Portuguesa, 1300-125 Lisboa, Portugal; rmartins@esscvp.eu (R.M.); tatalaia@esscvp.eu (T.A.)

**Keywords:** postural assessment, musculoskeletal disorders, anatomical metric analysis, machine learning, computer vision

## Abstract

From an early age, people are exposed to risk factors that can lead to musculoskeletal disorders like low back pain, neck pain and scoliosis. Medical screenings at an early age might minimize their incidence. The study intends to improve a software that processes images of patients, using specific anatomical sites to obtain risk indicators for possible musculoskeletal problems. This project was divided into four phases. First, markers and body metrics were selected for the postural assessment. Second, the software’s capacity to detect the markers and run optimization tests was evaluated. Third, data were acquired from a population to validate the results using clinical software. Fourth, the classifiers’ performance with the acquired data was analyzed. Green markers with diameters of 20 mm were used to optimize the software. The postural assessment using different types of cameras was conducted via the blob detection method. In the optimization tests, the angle parameters were the most influenced parameters. The data acquired showed that the postural analysis results were statistically equivalent. For the classifiers, the study population had 16 subjects with no evidence of postural problems, 25 with mild evidence and 16 with moderate-to-severe evidence. In general, using a binary classification with the train/test split validation method provided better results.

## 1. Introduction

The human being is, from an early age, exposed to high levels of stimulus and constrictions. Some can lead to musculoskeletal pain and disorders, such as neck and lower back pain or scoliosis. Postural defects are a growing problem that is increasingly affecting school-age children and adolescents [1]. A person’s posture changes throughout their life, with the greatest changes occurring during the period of growth [1]. It is in this period that a person’s bone structure develops, and sometimes the growth of the muscles and tendons does not keep up with the rate of bone growth, leading to greater rigidity in the joints which can lead to disorders and postural defects and cause orthopedic and rheumatologic diseases [2,3]. Additionally, over the course of time, postural defects can lead to pain complaints, which frequently limit daily physical activity [1].

Low back pain (LBP) and neck pain (NP) are pain and discomfort localized in the lumbosacral region and cervical region, respectively [4,5,6,7]. LBP and NP are symptoms frequently present in people of all ages and are increasingly common in school-aged children. Considering low back pain (LBP), one of the most common and expensive health care disorders in adulthood in industrialized countries [4,8,9,10], it frequently has its onset in adolescence; adult rates are reached by the age of 22 years [4,5,11], with evidence of implications for quality of life [4,9,12]. The prevalence of LBP in school-age children is high [13], reaching from 21% to 42% of adolescents who report episodes of LBP [14]; its rates rise until adulthood, with an estimated yearly prevalence of 20% and a lifetime prevalence of 70–80% [6,7,11,13,15,16]. Furthermore, studies show that musculoskeletal pain, specifically NP and LBP, in childhood and adolescence is a significant risk factor for experiencing such symptoms in adulthood and the possibly of the pain becoming chronic [4,6,14,15,16,17,18].

In a European survey [19], the prevalence of chronic pain at different body sites was assessed, and the results showed that the back in general had the highest prevalence, with 24% of the respondents reporting back pain without specifying the location, 18% reporting lower back pain, 8% reporting neck pain and 5% reporting upper back pain. Additionally, in a study by Dianat et al. [5], 59.6% of the population in the study (schoolchildren aged 12–14 years) reported neck, shoulder or low back pain during the month preceding the study. Of this 59.6%, low back, neck and shoulder pain were reported by 33%, 35.3% and 26.1% of the students, respectively.

Scoliosis is one of the most common spinal disorders in children and adolescents which also results in high costs to society; the causes can be idiopathic, related to joint hypermobility or due to postural behavior, among others [1,20,21,22,23]. Scoliosis refers to a three-dimensional structural deformation of the spine that involves a curvature in which a lateral flexion (known as the Cobbs angle) of more than 10° is formed in the spinal rotation in the frontal plane. The most common type of scoliosis is adolescent idiopathic scoliosis (AIS), and its onset frequently occurs after the start of puberty [20,21,22,24,25,26,27]. According to S. Bozkurt et al. [21], the prevalence of adolescent idiopathic scoliosis ranges from 1 to 3% among children and adolescents aged 10 to 16 years.

This disorder is accompanied by several symptoms, such as back pain, a deformation of the thoracic cage, a weakening of the respiratory muscles and a limited range of motion, and it can also cause reduced control over balance and gait asymmetry, therefore subsequently resulting in a reduced quality of life [20,26,28]. Mahaudens et al. [29] revealed that AIS patients need to perform 30% more physical effort than normal to ensure habitual locomotion, which requires an increase in oxygen consumption.

In most cases, these symptoms are due to non-specific causes, but there may be some risk factors that influence their onset [4,5,14,18,30], such as the experience of pain, being female, increased age, the intensity and frequency of exercise activities, poor posture adopted during the day, and the type of weight and the method of carrying the bag used in school [1,3,13,17,31]. The identification of these risk factors can facilitate the application of proper prophylactic actions to reduce pain and the limitation of physical and motor capabilities [1,15].

One proposed cause for LBP is impaired motor control; more specifically, altered postural control [32,33]. Correct postural control is important for performing daily activities [32,33,34], and includes a good development of dynamic balance and static postural control [34] and allows for adaptation when altering one’s posture and maintaining balance without running the risk of compromising performance or becoming injured [33,34,35]. The development of postural control happens especially at the age of 6–10 years [34]. Additionally, i people with LBP who have altered lumbosacral proprioceptive acuity and impaired trunk muscle control have been noted [33]. Furthermore, altered motor control has been proposed as a factor contributing to the persistence of pain; in addition, an association between the intensity of pain and the magnitude of postural sway has been proposed [35]. Also, idiopathic scoliosis seems to be associated with balance dysfunction. The severity of the postural imbalance may be associated with the progression and type of the curve, body posture and spinal deformity [36,37,38].

In addition to the high prevalence rates of these conditions, they are also important issues for national health services because they are some of the most expensive health care disorders with constantly increasing costs, especially LBP [4,23]. Regarding these musculoskeletal disorders, the indirect costs tend to be higher than the direct costs. In these situations, the indirect costs include a loss of ability to function in daily life, a loss of work productivity, sick leave and early retirement disability pensions. In adolescents, this implies an increase in school absenteeism and physical inactivity [9,12,14,39].

In 2019, 23.8% of the European Union’s population reported LBP or other chronic back problems. Also, 16.9% of the European Union’s population reported NP or other chronic neck problems [40]. In Portugal in 2020, around 123 thousand people had their work lives affected due to bone, joint or muscle problems which mainly affected the back. Also, around 94 thousand experienced bone, joint or muscle problems which mainly affected the neck. These data were collected during the second term of 2020, which was a period of lockdown [41].

Despite the rates of incidence of these pathologies, consultations are not very common. According to Gunzburg et al. [42] and Watson et al. [15], approximately only one-quarter of the school-aged children who reported LBP sought medical help.

The diagnosis of a musculoskeletal disorder is based on the use of medical imaging methods, such as X-ray and MRI, or postural assessment methods [27,43,44]. Given that most cases do not have an identified organic cause, postural analysis software and multidisciplinary pain treatment programs have been used increasingly [6,7,13,45]. However, these methods are used for diagnosis and for research purposes and are usually limited to health professionals in clinical environments with high associated costs [46,47].

Recently, mobile health apps (mHealth) became available for a small fee to the common user who suffers from low back and neck pain. These apps allow the user to be an active participant in maintaining or improving their health [48]. Nonetheless, they are generally intended to be used only after the onset of musculoskeletal pain in patients as a means of diagnosis or treatment.

It is important to prevent recurrent episodes of pain, chronicity and functional decline in individuals who suffer from musculoskeletal disorders as a way of improving their quality of life [12]. The early detection of musculoskeletal disorders during childhood might decrease their probability in adulthood or their likelihood of progressing to a chronic state as it allows children and adolescents to obtain help from a health professional.

The overall objective of this project was to modify and improve a preliminary version of a software created in an academic environment to be operable via mobile phone which processes images of a patient’s static posture and calculates various lengths and angles between body segments to provide risk scores for low back pain, neck pain or scoliosis to refer the child to a more specialized analysis conducted by a health professional. Based on the software that already existed, we determined four small objectives: to determine the best setup, anatomical markers and metrics necessary for the postural assessment and to enable the analysis of images captured via different types of cameras and scales. Subsequently, software optimization and the validation of the results were carried out using a non-pathological population.

## 2. Materials and Methods

The methodology used in the project was divided into four major phases: the improvement of a software already developed in an academic environment; the assessment of the software’s performance; the acquisition of data from an experimental group; and the implementation of classifiers. Each of these phases was also divided into several progressive steps. This project was approved by the ethics committee of ESSCVP–Lisboa (No. 09/2021).

### 2.1. Software Improvement

The software generated uses specific anatomical sites that need to be identified via markers to measure various anatomical parameters with the purpose of evaluating the subject’s posture.

**Figure 1:**
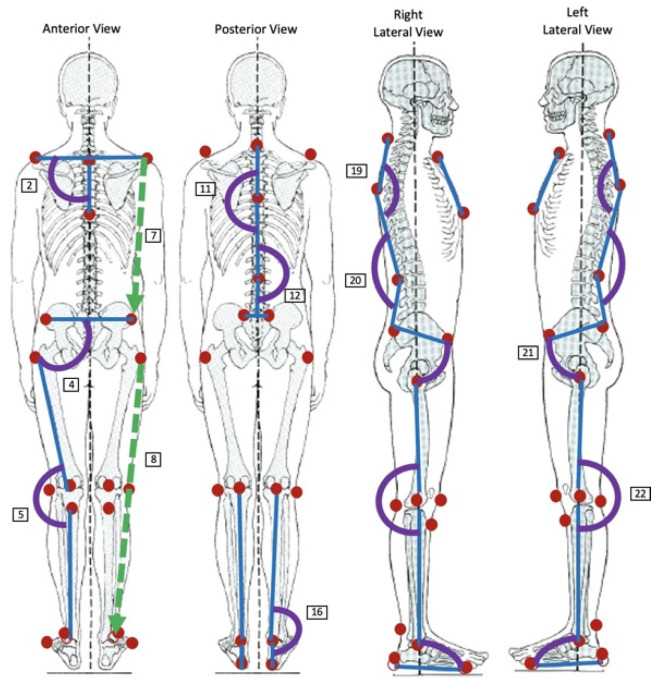
Anatomical sites selected for placing the markers (in red) and examples of the angles (in purple) and distances (in green) between body segments that the software will calculate in the posterior, anterior and the right and left lateral views.

In the first phase, the anatomical sites where the markers are placed were selected for all the different views (anterior, posterior and left and right lateral views), namely, the jugular notch, the xiphoid appendix, the spinous processes of C7, the most prominent point of thoracic kyphosis, the deepest point of lumbar lordosis, the acromion, the ASIS, the PSIS, the greater trochanter of the femur, the lateral condyle of the femur, the popliteal fossa, patella, tibial tuberosity, lateral malleolus, the posterior midpoint between the lateral and medial malleoli, the calcaneus and the fifth metatarsal. The last 12 anatomical sites mentioned are bilateral. These anatomical sites are presented in Figure 1 and were chosen in conjunction with physiotherapists and based on scientific articles about other postural analysis software [45,49,50].

In the second phase, we improved the code of the previously created program. We included the new marker positions selected and the anatomical parameters that the software should measure, such as the angles and the lengths of the body segments. Based on these values, the software produces scores of the risk indicators as an outcome. The software determines a total of 38 features, 15 of which 15 bilateral. These parameters are described in Table 1, and a list of their abbreviations is provided in the Abbreviation section. The markers and some of the angles and distances that are calculated are also represented in purple and green lines in Figure 1, respectively.

We also modified the code to allow the software to detect markers in images taken using different types of cameras, using the blob detection method. One was the camera of a mobile phone, an iPhone 8, and the second was a photographic camera, a Canon EOS 40D. These camera models were the same throughout all phases of this project.

The blob detection method consists of detecting regions in digital images that contain connected pixels that share the same light properties and are brighter or darker than the surrounding region; these regions are called blobs [51,52]. In this project, the color property of the pixels was also considered, and before applying this method, a threshold was applied to isolate the regions with colors of interest in the image. This method uses the properties of light for the detection of blobs and can also filter the identification of these regions through additional parameters such as area, circularity, convexity and the ratio of inertia. For each one of these, it is possible to define maximum and minimum values which are different from those defined by default in order to reduce the number of possible regions to be identified by the method [53].

The area parameter allows one to select the minimum and maximum area values that the detected region may have, making it possible to ignore regions that, despite having the desired color property, do not have an area within the desired range. Regarding the circularity parameter, it varies between 0 and 1 by default. The convex hull of a shape is the minimum convex set that contains all the points of that shape, and in turn, the convex set is a region in which all points on a line segment lie entirely within that region [54,55]. The ratio of inertia, which also varies between 0 and 1, defines how elongated the blob is. When this value is 1, it means that the region is a circle; however, as it approaches to 0, the region is shaped like an ellipse until it reaches the shape of a line that corresponds to the value 0 [53].

To obtain the scale of the image, we decided to use a rectangle with a known length which is placed on the left side of the image during image acquisition. In addition, to identify the line vertical to the floor, we used a string attached to the wall with a weight at its bottom end, as will be seen in the results.

### 2.2. Software Optimization

After selecting the set of markers, we understood which were the best marker characteristics that allowed the software to detect the markers better. Using a mobile phone camera, we obtained images of four subjects with different heights and volumes using different color combinations of clothes (white, brown, red, blue, green, yellow and gray) while using markers of different colors and sizes. To assess the size of the markers, we used white ping-pong balls with diameters of 40 mm and white Styrofoam balls with diameters of 20 mm. The 40 mm markers were the ones used in the previous version of the software. After selecting the best size, we assessed the best marker colors, using 20 mm Styrofoam balls 20 mm in the colors white, yellow, orange, red, blue and green. Each participant used all the 29 markers previously selected, and we obtained images of the anterior, posterior and left and right lateral views of the subject with and without flash. In this step, we also defined the colors of the rectangle and the string with a weight at the bottom.

Afterwards, we also evaluated the ability of the software to detect markers of colors different from the background with and without flash. We evaluated the colors white, brown, green, red, black, gray and blue as backgrounds. In this step, we placed the markers on a dress form. We also analyzed the ability of the software to detect the rectangle and the string with a weight at the bottom when using these backgrounds. For this step, we used the mobile phone and the photographic camera to assess the influence of the type of camera on the software’s perception of the image’s color.

In the next step, we analyzed the influence on the calculation of the angles and the distances between markers of varying the camera’s height, the camera’s distance to the wall, the use or lack of use of flash and the use of two types of cameras. In this step, we also placed a dress form with the markers in the same positions in front of the wall. We obtained pictures with the camera at different distances from the wall (250 cm). For each distance, we obtained pictures with the camera at different heights (90 cm and 110 cm), with and without flash and using the two types of cameras. The distances from the wall and the camera height were measured considering the center of the camera lens as the reference point. The dress form was used to guarantee that the markers were always in the same position in all the photos.

### 2.3. Data Acquisition

After completing the software optimization phase, we acquired image data to be assessed via our software and software Templo^®^ in the system Contemplas, version 13.1.654, in order to validate the results calculated using our software via a statistical analysis.

We prospectively collected data in the ESSCVP-Lisba laboratory facilities from a population composed of students from ESSCVP-Lisboa to obtain an initial database for the next phase of testing. The phase of data acquisition followed the Strengthening the Reporting of Observational Studies in Epidemiology (STROBE) guidelines.

Although the objective was to apply this software to the diagnosis of children and adolescents, we tested the adult students of ESSCVP-Lisboa as a first population. We chose these students because they were all adults, thus avoiding the operationally heavy process of obtaining consent from children’s parents. The data acquisition was performed during COVID-19 restrictions and confinement measures, which caused the process of collecting images and data from the participants to take longer than expected and did not allow us to consider the use of an experimental population composed of more participants.

#### 2.3.1. Description of Participants

The experimental group included a total of 60 participants who were only considered eligible patients if they were over the age of 18 years and were university students at ESSCVP-Lisboa. Being a student at ESSCVP-Lisboa was necessary due to logistics since the data acquisition was performed during COVID-19 restrictions and confinement measures. However, we only selected 57 students due to errors in the acquisition of images from three of the participants.

The participants were asked about their age, biological gender, height and weight; if they had pain in at least one of the cervical, dorsal and lumbar regions; if they went to a health professional regarding musculoskeletal pain and if they had previously been diagnosed with some type of musculoskeletal disorder.

After the data acquisition, each participant was divided into the following groups: without evidence of postural problems; with mild evidence; and with moderate-to-severe evidence.

#### 2.3.2. Materials and Experimental Setup

Each subject stood in front of a wall, with a 20 cm blue rectangle on the left side of the image aligned with the subject and a red wire with a weight on its end placed on the wall on the right side of the image. For each subject, four pictures of each view (anterior, posterior and lateral left and right) were taken, with green Styrofoam markers placed on the previously mentioned anatomical sites using double-sided tape.

#### 2.3.3. Technical Validation

After the phase of image acquisition, the images were analyzed using our software and the Contemplas software (which is a clinical software used for posture, motion and gait analyses). The Contemplas software was used to calculate the same parameters that our software calculates, using the markers at the same anatomical sites, for a posterior comparison and validation of the data calculated. The Contemplas software also provided a posture evaluation the posture. We used this software as a comparison since it was already used for postural assessments at the ESSCVP–Lisboa.

The Contemplas, an example of a PAS, is a computer software intended for use by professionals in a clinical environment. This software analyzes posture, movement or gait through images or video. Regarding the postural analysis, the software allows one to obtain a 2D or 3D assessment of static posture in which it measures the body axes, angles and measurements. In a 2D analysis, the software analyzes images from the posterior and side views. In a 3D analysis, an evaluation of an image from the anterior view is added and requires a minimum of 17 markers, though it is possible to use more markers to obtain additional metrics [56].

To compare the values obtained, we tested the difference in the significance between the means for each parameter calculated using both software programs. All the statistical hypothesis tests mentioned were carried out with significance level thresholds of 0.05 and 0.1.

Firstly, we separately analyzed the normality of the data representing each feature of each software to understand whether we should use a parametric or non-parametric statistical test to compare the means. To evaluate the normality of the data we used the Shapiro–Wilk test [57].

Next, we proceeded to compare the means for each pair of features. We applied the paired t-test if the feature data from both software programs were parametric; otherwise, we applied the Wilcoxon signed-rank test [58]. If a pair of features was significantly different for significance levels of both 0.1 and 0.05, we removed the outlier differences for those features and repeated the analysis.

Since these statistical hypothesis tests only allow us to know whether we can reject the null hypothesis or not but not whether we can accept it, we also applied an equivalence test for each pair of data [59,60]. The equivalence statistical test allows us to accept the alternative hypothesis that two data samples are similar to each other if the mean of the differences lies between two predefined boundaries. We first used the values 0.01 and 0.01 as lower and upper boundaries. If the test result was to not accept the alternative hypothesis, we increased the boundaries of the values, first to 0.05 and 0.05 and then to 0.06 and 0.06.

Afterwards, we presented the images to physiotherapy specialists at ESSCVP-Lisboa to evaluate the posture of the participants without the specialists knowing whether any of the patients had previously been diagnosed with a musculoskeletal disorder. The specialists also did not have previous access to the results obtained via our software or the Contemplas software. The assessment was compared with the Contemplas results to classify the postures in the images that were ambiguous and difficult to classify assertively. Considering the variables that our software analyzed, the participants’ postures were labelled as follows: without evidence of postural problems; with mild evidence of postural problems; and with moderate-to-severe evidence of postural problems.

### 2.4. Classification

After validating the results obtained via our software, we implemented classifiers which used the anatomical parameters calculated to indicate the existence or absence of evidence of postural problems.

First, we performed a feature selection to reduce and select the best features to apply in the classifiers to, using the Orange toolbox. To select the best set of features, we ranked the features using the Information Gain method, the Chi-squared method and the Relief method.

Second, we implemented two different widely used algorithms to train the classifier: a Linear Discriminant Analysis (LDA) and k-Nearest Neighbors (kNN) [61]. For each classification model, we used two validation methods to test the dataset: 10-fold cross-validation and a train/test split with 70% training and 30% testing [62,63].

**Figure 2:**
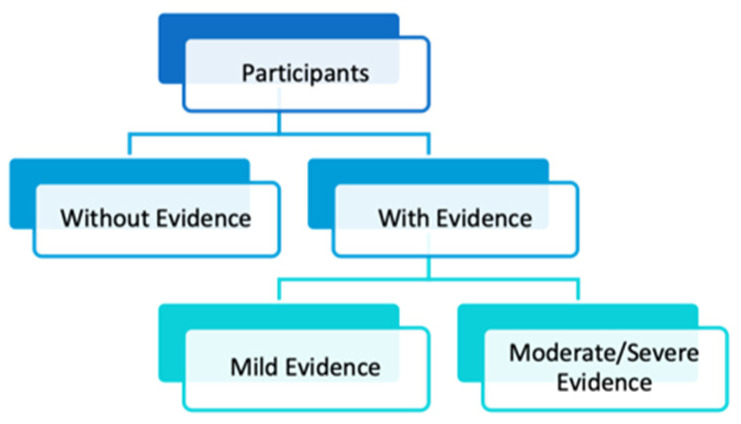
Diagram of the two-level binary classification.

We started by considering a three-class multiclass classifier, labeling the 57 participants as follows: no evidence of postural problems, mild evidence of postural problems and moderate-to-severe evidence of postural problems. Then we considered a two-level binary classification to understand whether the results obtained would be better. The first level classified 57 subjects between no evidence and evidence of postural problems. The second level divided the 41 subjects with evidence into the following categories: subjects with mild evidence and subjects with moderate-to-severe evidence. The two-level binary classification is exemplified in the diagram in Figure 2.

When applying the kNN model, we first obtained the error rate values corresponding to each value of k. We chose the five values of k with which we obtained the lowest error rates. We then tested the kNN model for each of the five chosen values of k and compared the results obtained with these models and with the LDA model.

## 3. Results

### 3.1. Software Optimization

In the analysis for the best marker characteristics, we first compared a white ping-pong ball marker with a diameter of 40 mm and a white Styrofoam marker with a diameter of 20 mm. We accessed the rate of the detection of the markers by the software; this was the number of markers detected by the software, which is shown in Table 2.

After, we considered a Styrofoam marker with a diameter of 20 mm, and we compared the use of markers of different colors. For this, we considered a success rate, which was the number of real markers with approximately 100% of their areas detected. The results of the success rate for each colored marker are shown in Table 3.

In the last step of the software optimization, we observed the influence of the use or not of flash, the camera’s distance to the wall, the camera’s height and the use of two different types of cameras on the results of calculating the anatomical parameters.

**Figure 3:**
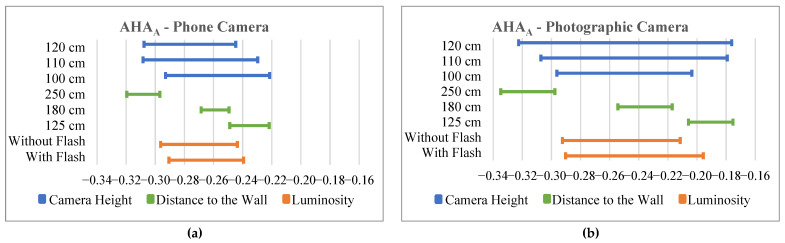
Results of the 95% confidence interval for the mean obtained in the AHA_A_ calculation when using the mobile phone camera (**a**) and when using the photographic camera (**b**). The values in the horizontal axis are angles in degrees.

**Figure 4:**
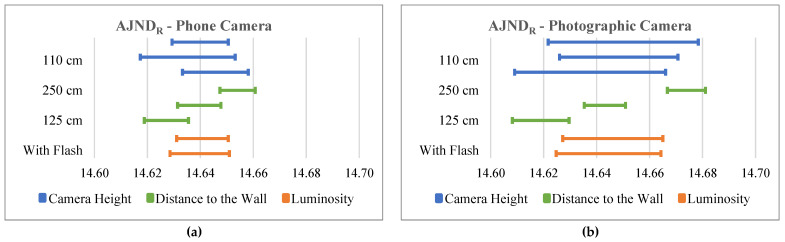
Results of the 95% confidence interval for the mean obtained in the AJND_R_ calculation when using the mobile phone camera (**a**) and when using the photographic camera (**b**). The values in the horizontal axis are distances in cm.

To better understand the results, for each anatomical parameter, we created a graphic comparing the 95% confidence interval for the mean of each of the three distances to the wall, each of the three camera heights and of the use or not of flash while using a phone camera. Another similar graphic was created for the use of the photographic camera for the same anatomical parameters. In Figure 3, we show an example of this pair of graphics for an anatomical parameter that is an angle (AHAA). An example pair of these graphics for an anatomical parameter that is a distance (AJNDR) is shown in Figure 4.

### 3.2. Comparison with the Contemplas Software

We used the images acquired with the study population to validate the results from our software and compare them with the Contemplas software. The demographic characteristics of the study population are shown in Table 4.

In addition to the previous demographic variables, we also determined that of the 57 subjects, 30 claimed to have pain in at least one of the cervical, dorsal and lumbar regions. Of these thirty people, only eight visited a health professional, four of which were diagnosed with a pathology prior to the study.

To validate our data, we first compared the difference between the means with significance levels equal to 0.05 and 0.1. Most of the pairs of anatomical parameters were not statistically significant. Only the pairs of parameters ALLL_L_ and PSISHA were statistically significant, at least when α = 0.1. After removing the outliers of these two pairs, the result was not statistically significant for either significance level used. The results obtained during the statistical analysis of these two pairs of parameters are presented in Table 5, which shows the mean, standard deviation and extreme values of the data, as well the *p*-value analysis.

After, since we wanted to better demonstrate that the data calculated with both software programs were similar, we also performed an equivalence test for all the pairs of parameters. We considered ±0.01 and ±0.05 as boundaries and the significance levels of 0.05 and 0.1. All pairs had a statistically significant equivalence when the boundaries were ±0.01; however most of them were equivalent with the boundaries of ±0.05. Only the pair of anatomical parameters ALLL_L_ and PSISHA required that the boundaries be increased to ±0.06 to be significantly equivalent when the significance level was 0.05, as demonstrated in Table 6.

### 3.3. Classification

After comparing the results obtained via both software programs, the posture of each participant was assessed by physiotherapy specialists using the acquired images. With the specialist assessment and the Contemplas software evaluation, we considered that the study population had 16 subjects without any evidence of posture problems and 41 who presented evidence of posture problems. Of these 41 people, 25 presented mild evidence of posture problems and 16 presented moderate-to-severe evidence of posture problems.

#### 3.3.1. Feature Selection

We first performed a feature selection considering a multiclass classifier which selected the following set: ASA, AJND_L_, AAD_L_, AAD_R_, PSISHA, PSISLA_R_, APD_L_, APD_R_ and LLC_L_.

Afterwards, we also selected the best set of features to implement both levels of the binary classifier. The set selected for the first level was AHA_A_, ASA, AAD_L_, TKA, LLA, PKLA_L_, PKLA_R_, APD_L_, APD_R_, PLLL_R_, TKC_L_ and LLC_L_. For the second level, we selected the set ASISLA_R_, AKLA_R_, AAD_L_, AAD_R_, APD_L_, APD_R_ and LLC_L_.

#### 3.3.2. Classification Performance

The performance of the classifiers was assessed using the accuracy and f1 scores of each class. Table 7 summarizes the accuracy and f1-score results obtained when using the LDA and the kNN models with both the 10-fold cross-validation and the train/test split validation methods in the multiclass classification and level 1 and level 2 of the binary classification.

Only the results related to the five kNN models selected in each analysis are presented and compared with the other results.

## 4. Discussion

### 4.1. Software Optimization

The analysis of the two different marker sizes showed that the 40 mm marker had a slightly higher detection rate. However, one-quarter of the 40 mm markers had only part of their area detected due to shadows created by the other markers. Considering the success rate to be the number of real markers with approximately 100% of their areas detected, we obtained a higher success rate using the 20 mm markers.

After selecting 20 mm as the marker size the markers, we evaluated the best marker color the marker. While using the white and yellow colors, the software confused the marker’s color with the background and did not detect some of the markers. The use of orange and red caused the detection of false markers on the skin of the subjects. We obtain the highest success rate with green, which was selected as the marker color.

None of the colors used achieved a 100% success rate since some markers were not detected because they were partially or completely covered by body parts or folds of clothing.

One hypothesis to study in the future is the consideration of multi-color markers with a specific arrangement to reduce confusion with the background and the subject’s clothes. With more than one color in the marker, we could identify the color of the background and only threshold the other colors in the multi-color marker. In this way, we could detect the marker even if a portion of the marker has the same color as the background or the subject’s clothes.

Regarding the size of the markers, in this study, we first used the 40 mm markers since this was the size previously considered. We also decided to test the 20 mm markers to try to reduce the diameter; this allowed us to reduce it by half while still maintaining the ease at which the software detected the markers. For future studies, we should reduce the size of the markers to obtain more precise and useful data.

We also opted to use blue for the rectangle used to obtain the image scale and red for the string with a weight at its end to know what is the vertical to the floor. We choose different colors for the three objects to ensure that the software would not confuse them.

The assessment of the software’s ability to detect these three objects using different backgrounds colors showed that the markers and the rectangle were correctly detected against all backgrounds. The red string was not detected only when the red background was used.

In the last step of the software optimization, we observed that the variable that least influenced the results was the use or not of flash.

The variation in the camera’s distance to the wall resulted in the intervals with the smallest amplitudes; however, their mean values had the greatest distances between them. That is, for the same distance, the values calculated were similar, but the mean value obtained at different distances was the most different. Furthermore, the increase in the camera’s distance from the wall showed a slight decrease in the angle parameter values and an increase in the distance between the marker parameters.

Regarding the camera-height variable, the results showed the greatest interval amplitude of all the variables. This variable had more influence on the angle parameters.

When comparing the types of cameras used, we verified that when using the photographic camera, the results showed greater variation than when a cell phone camera was used.

These variations might be due to the rectangle and the string not being exactly in the same plane as the markers, leading to parallax errors. Another possible reason is that the camera lens was not positioned completely parallel to the dress form. A possible solution is to obtain the vanishing point of the image, allowing these objects to be placed in different planes. Another possibility is to know the size of the camera sensor through the image specifications, allowing one to obtain more exact results which are similar between different types of cameras.

### 4.2. Comparison with the Contemplas Software

In order to validate the results from our software, we compared them with the results obtained using the Contemplas software. We conducted a statistical analysis to compare the difference between means, which showed that most of the pairs of the same parameter were not significantly different for both significance levels tested. Two pairs of features (ALLL_L_ and PSISHA) had low *p*-values; thus, we removed the outliers. Removing the outliers resulted in *p*-values that were higher than both significance levels, meaning they were not significantly different. However, not being significantly different does not mean that we could accept the null hypothesis, so we also performed an equivalence of means hypothesis test to evaluate whether the difference between means fell inside two pre-selected boundaries. Neither of the pairs was significantly equivalent between ∆L = −0.01 and ∆U = 0.01; however, most of the pairs of features were significantly equivalent between ∆L = −0.05 and ∆U = 0.05 at both significance levels. Two pairs of features (ALLL_L_ and PSISHA) were only significantly equivalent at a 5% significance level when the boundaries were ∆L = −0.06 and ∆U = 0.06. Therefore, at this point, we could consider that each pair of features was significantly equivalent.

During the image analyses, we noticed that some markers were not fully colored green or were partially covered by body parts, especially in the back area in the lateral-view images. Therefore, we had to manually draw green circles on these markers with the same dimensions and approximate location. For future postural assessments, we need to correct this problem, possibly by moving the markers away at the same distance from the body while maintaining the angles between them. This solution would mainly be applied to the markers located on the back.

We could also test a more simplified setup, using a set square with a weight on one of the edges, replacing the blue rectangle and the red string by having just one object with both functions.

The demographic data regarding postural problems were initially collected in order to attain an idea of the existence of evidence or the diagnosis of postural problems in the study population. We would use this last type of information to classify the subjects’ posture. However, few subjects answered that they had visited a health professional because of these disorders, and there was not enough data to be used for this purpose. On the other hand, these values also show that less than 30% of the study population who suffered from back pain sought medical help. In comparison with the value of approximately only one-quarter of school-aged children who reported LBP and sought medical help [15,42], we can observe an increase of 5%. This difference in the values could be possible explained due to our population being composed of adults who are possibly already more aware of musculoskeletal problems and their consequences.

### 4.3. Classification

We implemented the LDA and the kNN classifiers, each using the validation methods of a 10-fold cross-validation and a train/test split with 70% training and 30% testing. First, we considered a multiclass classification with classes; then, we considered two binary classifications.

In the results of the multiclass classification, we observed that the highest accuracy value (61.11%) was obtained when we used the kNN algorithm with k = 9, and the lowest accuracy value (50%) was obtained when we used the LDA model, both while using the train/test split validation method. Regarding the f1-score values, the values of the “mild evidence” class had the highest values of the three classes with both validation methods. The LDA models were the models with the lowest differences between the f1-score values. The results of the f1-score values also showed lower differences between the classes when using the 10-fold cross-validation method than when using the train/test split validation method.

Observing the results of level 1 of the binary classification, we verified that the values in the class “with evidence” were higher than the values of the “without evidence” class. The kNN model which used k = 15 and the train/test split validation method showed the best results of the three metrics analyzed in this step (accuracy = 83.33%, f1-score “without evidence” = 66.67% and f1-score “with evidence” = 88.89%). Also, in this classification, both LDA models had the lowest accuracy values.

In the results of level 2 of the binary classification, the “mild evidence” class had higher f1-score values than the “moderate/severe evidence” class. Once again, the LDA models had the lowest accuracy values for both validation methods, as well as the lowest f1-score values of the two classes. The model with the highest results for the three metrics was the kNN model when k = 6 and k = 7 and when using the train/test split validation method.

In both levels of the binary classification, the overall results while using the train/test split validation method were higher than when the 10-fold cross-validation method was used. Although the use of the 10-fold cross-validation method usually makes the classifier more robust, these results can be explained by the small size of the database since, with 57 participants, each fold is composed of approximately 6 participants, which can lead to bias in the distribution of the data. In future projects, a larger study population should be used so that each fold can be composed of a larger sample size to train the classifiers.

The overall results of the kNN method confirm that it helps decrease the false positive rate, thus increasing the overall accuracy of the model [64].

When comparing the results obtained using the multiclass classification and with both levels of the binary classification, we verified that the binary classification had, in general, better values and less confusion between classes than the multiclass classification. This result is in line with the statement that when dealing with a multi-class problem, the decomposition of the original problem into a set of binary subproblems is an easy yet accurate way to reduce complexity [65,66].

During the course of this study, some limitations were identified, as mentioned during this discussion. In the future, it will be necessary to correct them, namely, to improve the parallax errors inherent in image capture; to ensure that no marker is covered by body parts; to increase the number of participants in the study population; and to test the software in a population comprising children and adolescents, who are the target audience, in order to generate a database that is better adapted to those ages.

Other steps to be taken are to finish developing the easy-to-use prototype kit that contains an adjustable harness with the markers stitched on. We will also need to turn the software, which is still in offline mode, into a mobile application that is associated with a secure server to store and process the data. The final objective is to implement the use of this kit in schools and youth sports associations, encouraging screening from an early age and allowing for the referral of children to more specialized analyses by health professionals.

## 5. Conclusions

Musculoskeletal pain and disorders, such as LBP, NP and scoliosis, are common in adulthood. On a daily basis, these disturbances affect the quality of life of populations and can have consequences on people’s professional lives. Their early detection during childhood and adolescence might reduce the likelihood of LBP, NP and scoliosis in adulthood or of these disorders becoming chronic.

Despite some limitations, we have made some advances in the development of our software. We made an automatic analysis of images from different cameras possible, and we achieved an understanding of the best conditions for obtaining an image. We found the best set of points and anatomical features for assessing the subjects’ posture. We validated the results obtained via our software with a study population, and we implemented classifiers as parameter evaluators and not as final diagnostic classifiers.

This study made some advances in the development of this software. This work will contribute to the screening of musculoskeletal pain and disorders, such as LBP and scoliosis, in children and adolescents, and might allow for a decrease in the probability of their appearance in adulthood or of their progression to a chronic state.

## Figures and Tables

**Table 1:** Anatomical parameters calculated by the software and respective descriptions of the anatomical points used.

	Parameter	Description of Anatomical Landmarks Used
Anterior	1. Acromions Horizontal Alignment (AHA_A_)	Angle between the two acromions and a horizontal line
	2. Acromions–Sternum Angle (ASA)	Angle between the jugular notch and the xiphoid appendix and the two acromions’ line
	3. ASISs Horizontal Alignment (ASISHA)	Angle between the two ASISs and a horizontal line
	4. ASISs–Leg Angle (ASISLA_L_; ASISLA_R_)	Angle between the trochanter and patella and the line of the two ASIS’s line
	5. Knee Lateral Angle (AKLA_L_; AKLA_R_)	Angle between the line of the trochanter and the patella and the line of the tibial tuberosity and the lateral malleolus
	6. Acromion–Jugular Notch Distance (AJND_L_; AJND_R_)	Distance between the jugular notch and each acromion
	7. Acromion–ASIS Distance (AAD_L_; AAD_R_)	Distance between the acromion and the ASIS of the same side
	8. Lower Limb Length (ALLL_L_; ALLL_R_)	Distance between the trochanter and the lateral malleolus of the same side
Posterior	9. Acromions Horizontal Alignment (AHA_P_)	Angle between the two acromions and a horizontal line
	10. Acromions-Vertebral Column Angle (AVA)	Angle between the C7 and the most prominent point of thoracic kyphosis and the line of the two acromions
	11. Thoracic Kyphosis Lateral Angle (TKA)	Angle between C7, the most prominent point of thoracic kyphosis and the deepest point of lumbar lordosis
	12. Lumbar Lordosis Lateral Angle (LLA)	Angle between the most prominent point of thoracic kyphosis and the deepest point of lumbar lordosis and the midpoint of the PSISs
	13. PSISs Horizontal Alignment (PSISHA)	Angle between the two PSISs and the horizontal line
	14. PSISs-Leg Angle (PSISLA_L_; PSISLA_R_)	Angle between the trochanter and popliteal fossa and the line of the two PSISs
	15. Knee Lateral Angle (PKLA_L_; PKLA_R_)	Angle between the trochanter and the popliteal fossa and the posterior midpoint between the lateral and medial malleolus
	16. Ankle Lateral Angle (ALA_L_; ALA_R_)	Angle between the popliteal fossa, the posterior midpoint between the lateral and the medial malleoli and the calcaneus
	17. Acromion-PSIS Distance (APD_L_; APD_R_)	Distance between the acromion and the PSIS of the same side
	18. Lower Limb Length (PLLL_L_; PLLL_R_)	Distance between the trochanter and the calcaneus of the same side
Lateral	19. Thoracic Kyphosis Curvature (TKC_L_; TKC_R_)	Angle between C7, the most prominent point of thoracic kyphosis and the deepest point of lumbar lordosis
	20. Lumbar Lordosis Curvature (LLC_L_; LLC_R_)	Angle between the most prominent point of thoracic kyphosis, the deepest point of lumbar lordosis and the PSIS
	21. Pelvis–Leg Angle (PLA_L_; PLA_R_)	Angle between the line of the ASIS and the PSIS and the line of the trochanter and the lateral femoral condyle
	22. Knee Angle (KA_L_; KA_R_)	Angle between the trochanter, the lateral femoral condyle and the lateral malleolus
	23. Leg–Foot Angle (LFAL; LFAR)	Angle between the line of the trochanter and the lateral femoral condyle and the line of the calcaneus and the fifth metatarsal

**Table 2:** Detection rate while using markers with 40 mm and 20 mm diameters.

Marker Size	Detection Rate
40 mm—Ping-Pong Ball	75.2%
20 mm—Styrofoam Ball	64.8%

**Table 3:** Success rate while using markers of different colors.

Marker Color	Success Rate
White	64.8%
Yellow	89.9%
Orange	95.4%
Red	93.9%
Blue	94.2%
Green	96.7%

**Table 4:** Demographic characteristics of the experimental population.

Age	Biological Gender (F/M)	Height (cm)	Weight (kg)
20.9 ± 4.5	39/18	166.4 ± 9.3	69.4 ± 16.3

**Table 5:** Results of the mean, standard deviation and extreme values before and after removing the outliers from the parameters ALLL_L_ and PSISHA acquired via the Contemplas software and via our software, in addition to the result of the *p*-value obtained in the statistical analysis to evaluate the difference in the means of a pair composed of the same parameters acquired via the two software programs.

			ALLL_L_	PSISHA
Before removing the outliers	Mean ± Standard Deviation	Our Software	68.65 ± 5.25	0.58 ± 2.72
		Contemplas	68.62 ± 5.25	0.55 ± 2.70
	Values Range	Our Software	[58.35; 80.05]	[−9.90; 5.04]
		Contemplas	[58.5; 80.0]	[−9.8; 5.1]
	*p*-Value		0.058	0.047
	Statistically Significant α = 0.05/α = 0.1		No/Yes	Yes/Yes
After removing the outliers	Mean ± Standard Deviation	Our Software	68.64 ± 5.3	-0.58 ± 2.76
		Contemplas	68.62 ± 5.3	-0.56 ± 2.74
	Values Range	Our Software	[58.35; 80.05]	[−9.9; 5.04]
		Contemplas	[58.5; 80.0]	[−9.8; 5.1]
	*p*-Value		0.01	0.02
	Statistically Significant α = 0.05/α = 0.1		No/No	No/No

**Table 6:** Results of the *p*-values obtained when applying the equivalence test to evaluate the equivalence between the means of the features acquired via the two software programs for the pair ALLL_L_ and the pair PSISHA.

		ALLL_L_	PSISHA
**ΔL = −0.01** **ΔU = 0.01**	*p*-Value	0.89	0.91
	Statistically Significant α=0.05/α = 0.1	No/No	No/No
**ΔL = −0.05** **ΔU = 0.05**	*p*-Value	0.05	0.07
	Statistically Significant α=0.05/α = 0.1	No/Yes	No/Yes
**ΔL = −0.06** **ΔU = 0.06**	*p*-Value	0.01	0.02
	Statistically Significant α=0.05/α = 0.1	Yes/Yes	Yes/Yes

**Table 7:** Results of the accuracy and f1-score values of each class, obtained via an analysis of the classifier’s performance.

	**Train/Test Split Validation (70%Train/30%Test)**						**10-Fold Cross-Validation**					
	Multiclass											
	LDA	k = 3	k = 4	k = 5	k = 6	k = 9	LDA	k = 4	k = 5	k = 6	k = 8	k = 12
Accuracy	50%	55.56%	55.56%	55.56%	55.56%	61.11%	56.14%	59.65%	59.65%	57.89%	59.65%	56.14%
F1-Score “Without Evidence”	36.36%	53.33%	53.33%	53.33%	53.33%	66.67%	43.75%	58.82%	57.89%	57.89%	57.89%	58.82%
F1-Score “Mild Evidence”	57.14%	76.92%	76.92%	76.92%	76.92%	70.59%	65.38%	65.38%	64%	62.75%	66.67%	63.33%
F1-Score “Moderate/ Severe Evidence”	54.55%	25%	25%	25%	25%	28.57%	53.33%	50%	53.85%	48%	48%	30%
	Binary—Level 1											
	LDA	k = 9	k = 13	k = 14	k = 15	k = 17	LDA	k = 8	k = 9	k = 10	k = 17	k = 21
Accuracy	72.22%	77.78%	77.78%	77.78%	83.33%	77.78%	63.16%	77.19%	77.19%	77.19%	77.19%	77.19%
F1-Score “With Evidence”	80%	84.62%	84.62%	83.33%	88.89%	85.71%	74.07%	83.12%	83.54%	83.12%	85.39%	86.02%
F1-Score “Without Evidence”	54.55%	60%	60%	66.67%	66.67%	50%	36.36%	64.86%	62.86%	64.86%	48%	38.1%
	Binary—Level 2											
	LDA	k = 6	k = 7	k = 9	k = 10	k = 11	LDA	k = 3	k = 4	k = 5	k = 6	k = 9
Accuracy	76.92%	92.31%	92.31%	84.62%	84.62%	84.62%	60.98%	70.73%	70.73%	70.73%	73.17%	73.17%
F1-Score “Mild Evidence”	84.21%	94.74%	94.74%	90%	90%	90%	69.23%	76.92%	79.31%	78.57%	81.36%	81.36%
F1-Score “Moderate/ Severe Evidence”	57.14%	85.71%	85.71%	66.67%	66.67%	66.67%	46.67%	60%	50%	53.85%	52.17%	52.17%

Note: All columns with an indication of a k-value refer to kNN classifiers. All the values in this table are percentages (%).

## Data Availability

Data supporting this study are not publicly available due to ethical reasons. Please contact the corresponding author.

## Abbreviations

Anatomical Parameters Abbreviation

AAD_L_	Left Acromion–ASIS Distance
AAD_R_	Right Acromion–ASIS Distance
AHA_A_	Acromions Horizontal Alignment in Anterior View
AHAP	Acromions Horizontal Alignment in Posterior View
AJND_L_	Left Acromion–Jugular Notch Distance
AJND_R_	Right Acromion–Jugular Notch Distance
AKLA_L_	Left Knee Lateral Angle in Anterior View
AKLA_R_	Right Knee Lateral Angle in Anterior View
ALA_L_	Left Ankle Lateral Angle
ALA_R_	Right Ankle Lateral Angle
ALLL_L_	Left Lower Limb Length in Anterior View
ALLL_R_	Right Lower Limb Length in Anterior View
APDL	Left Acromion–PSIS Distance
APDR	Right Acromion–PSIS Distance
ASA	Acromions–Sternum Angle
ASISHA	ASISs Horizontal Alignment
ASISLAL	ASISs–Left Leg Angle
ASISLAR	ASISs–Right Leg Angle
AVA	Acromions–Vertebral Column Angle
KA_L_	Knee Angle in Left Lateral View
KA_R_	Knee Angle in Right Lateral View
LFA_L_	Leg–Foot Angle in Left Lateral View
LFA_R_	Leg–Foot Angle in Right Lateral View
LLA	Lumbar Lordosis Lateral Angle
LLC_L_	Lumbar Lordosis Curvature in Left Lateral View
LLC_R_	Lumbar Lordosis Curvature in Right Lateral View
PKLA_L_	Left Knee Lateral Angle in Posterior View
PKLA_R_	Right Knee Lateral Angle in Posterior View
PLA_L_	Pelvis–Leg Angle in Left Lateral View
PLA_R_	Pelvis–Leg Angle in Right Lateral View
PLLL_L_	Left Lower Limb Length in Posterior View
PLLL_R_	Right Lower Limb Length in Posterior View
PSISH_A_	PSISs Horizontal Alignment
PSISLA_L_	PSISs–Left Leg Angle
PSISLA_R_	PSISs–Right Leg Angle
TKA	Thoracic Kyphosis Lateral Angle
TKC_L_	Thoracic Kyphosis Curvature in Left Lateral View
TKC_R_	Thoracic Kyphosis Curvature in Right Lateral View
